# Overcoming Hydrophobicity with Water Enables Ultrafast Hydrolysis of Waste Polyethylene Terephthalate at Very Mild Conditions

**DOI:** 10.1002/anie.202514136

**Published:** 2025-11-10

**Authors:** Francesco Millucci, Raimondo Germani, Leonardo Colelli, Serena Gabrielli, Paola Sassi, Anna Donnadio, Martina Conti, Silvia Corezzi

**Affiliations:** ^1^ Dipartimento di Fisica e Geologia Università di Perugia Perugia I‐06123 Italy; ^2^ Dipartimento di Chimica Biologia e Biotecnologie Università di Perugia Perugia I‐06123 Italy; ^3^ Department of Chemical Engineering Materials Environment “Sapienza” University of Rome Rome I‐00184 Italy; ^4^ Chemistry Interdisciplinary Project (ChIP) Scuola di Scienze e Tecnologie Università di Camerino Camerino I‐62032 Italy; ^5^ Dipartimento di Scienze Farmaceutiche Università di Perugia Perugia I‐06123 Italy; ^6^ CNR ‐ Istituto Officina dei Materiali (IOM) Area Science Park Basovizza Trieste I‐34149 Italy

**Keywords:** Alkaline hydrolysis, Chemical recycling, Hydrophobicity, Mild conditions, Water‐swollen PET

## Abstract

Chemical recycling of plastics holds great promise but remains constrained by sustainability issues, with polyethylene terephthalate (PET) epitomizing this challenge. Herein, we introduce a conceptually novel strategy that overcomes PET's intrinsic hydrophobicity by physically re‐engineering the polymer's microstructure to enable ultrafast alkaline hydrolysis under exceptionally mild conditions. We leverage the ability of propylene carbonate (PC)—an inexpensive, commercial, green solvent—to selectively dissolve PET, to thermally induce phase separation, and subsequently act as a carrier for water insertion between polymer chains. Upon complete PC replacement, the water uptake exceeds twice the polymer mass, preventing chain re‐compaction and establishing an interfacial environment that facilitates hydroxyl ion diffusion to ester bonds and depolymerization with minimal alkali consumption. As a result, water‐swollen PET fully depolymerizes (96% TPA yield) at atmospheric pressure within 5 min at 90 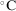 or under 2 h at room temperature, vastly outperforming conventional hydrolysis methods. The process achieves a >20‐fold reduction in energy footprint versus direct PET hydrolysis. It performs robustly on challenging, real‐world feedstocks—including textiles and mixed plastic waste—enabling selective depolymerization unaffected by PET crystallinity. A techno‐economic analysis (TEA) confirms energy efficiency and strong economic feasibility, demonstrating overall competitiveness with existing engineered technologies. Beyond PET, the physical mechanism underpinning the strategy offers a scalable and sustainable platform for recycling a wide range of condensation polymers.

## Graphical abstract

Entrapping water within the microstructure of hydrophobic PET unlocks ultrafast hydrolysis under remarkably mild conditions. The approach demonstrates a rare combination of high chemical efficiency, low environmental burden, and strong potential for scale‐up — significantly advancing the prospects for sustainable recycling of condensation polymers.

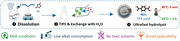

## Introduction

Due to their cost‐effectiveness, durability, and thermomechanical properties, plastics are indispensable to modern society, but their accumulation threatens ecosystems globally.^[^ ^1^, ^2^ ^]^ Polyethylene terephthalate (PET), widely used in the packaging and textile industries and known for its extremely slow natural degradation, has emerged as an iconic symbol of anthropogenic pollution. Currently, it is mainly recycled through mechanical processes, which often degrade its structural integrity and limit reuse to lower‐value products.^[^ ^3^ ^]^ These methods are also ineffective for recovering PET from mixed or contaminated waste streams. In response, chemical recycling offers a more circular and sustainable solution, by breaking down the polymer to its constituent monomers which can be repolymerized into plastic of virgin‐grade quality or used to produce value‐added materials.^[^ ^4^ ^]^

Depending on the nucleophilic reagent employed to cleave ester bonds, PET depolymerization can be achieved through various methods—ranging from conventional glycolysis,^[^ ^5^ ^]^ methanolysis,^[^ ^6^ ^]^ and hydrolysis,^[^ ^7^ ^]^ to more recent acetolysis^[^ ^8^ ^]^ and hydrogenolysis^[^ ^9^ ^]^—with hydrolysis standing out for using water—the greenest solvent on Earth—as the reaction medium, and for producing terephthalic acid (TPA), the raw material in most PET production lines in the petrochemical industry.^[^ ^10^ ^]^ Hydrolysis occurs in neutral,^[^ ^11^ ^]^ acidic,^[^ ^12^ ^]^ and alkaline ^[^ ^13^ ^]^ environments, as well as through enzymatic processes.^[^ ^14^, ^15^ ^]^ Among hydrolytic methods, alkaline hydrolysis offers the greatest potential for scale‐up, as it yields monomers of higher purity and avoids the pre‐amorphization step required by enzymatic processes.^[^ ^16^ ^]^ At least two alkaline hydrolysis‐based technologies are being commercialized in Europe: the microwave‐assisted GR3N process^[^ ^17^ ^]^ and the UV‐photocatalyzed DePoly process,^[^ ^18^ ^]^ both of which operate pilot plants and attract investors for scaling up.^[^ ^19^, ^20^ ^]^ These commercially relevant examples demonstrate that alkaline hydrolysis is a viable strategy for PET recycling. However, developing more sustainable pathways remains a key challenge, requiring the simultaneous fulfillment of critical demands such as accelerating the reaction under mild conditions, minimizing alkali consumption, and avoiding hazardous solvents and costly equipment.

Alkali‐promoted hydrolysis mainly relies on alkaline hydroxides such as NaOH and KOH, whose ratio to PET is one of the most critical factors,^[^ ^21^ ^]^ as it dictates the amount of acid required to neutralize the alkali and precipitate TPA. In pure aqueous solution, a stoichiometric amount of NaOH relative to PET (0.41:1 w/w) necessitates ≳1 h under harsh conditions (200 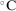; 1.5 MPa) to achieve TPA yields above 95%.^[^ ^13^ ^]^ Phase transfer catalysts have been used to allow milder conditions (90–100 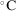; 0.1 MPa). However, good catalytic performances have been achieved either with longer reaction times,^[^ ^22^ ^]^ or with significant (fourfold) stoichiometric excess of NaOH in combination with ultrasound^[^ ^23^ ^]^ or microwave irradiation.^[^ ^24^ ^]^ Rapid catalytic hydrolysis at 138 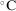 without the need for high‐pressure vessels and microwave equipment has required the use of NaOH at 12.5 M—an extremely corrosive concentration that severely limits scalability.^[^ ^25^ ^]^ Additionally, catalyst recovery increases the process's operational complexity. An approach to accelerate the reaction involves the introduction of co‐solvents. For instance, an optimized 95% TPA yield has been obtained in 20 min at 80 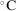 using 60 vol% ethanol in water.^[^ ^26^ ^]^ However, this yield requires a sixfold excess of NaOH and strongly depends on PET's crystallinity and specific surface area, dropping to 20% for particles >1 cm. Methods using non‐aqueous solvents have been developed to enhance PET solubility and regulate hydroxide reactivity.^[^ ^27^, ^28^, ^29^, ^30^, ^31^ ^]^ Although these methods may be fast or operate under mild conditions, they rely on volatile (e.g., methanol, ethanol, tetrahydrofuran) or toxic chemicals (e.g., dichloromethane), raising concerns about human health and environmental safety. The use of green solvents, such as γ‐valerolactone, for PET dissolution has also been explored,^[^ ^32^ ^]^ enabling accelerated alkaline hydrolysis under mild reaction conditions (8 min at 90 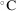). However, this benefit still comes at the cost of excessive alkali consumption (NaOH:PET = 3:1 w/w), leading to highly corrosive conditions that are undesirable for industrial implementation. Thus, no currently available pathway effectively integrates and balances the key requirements for sustainable hydrolysis.

In this work, we present a strategy to entrap an exceptionally large amount of water within hydrophobic PET, enabling ultrafast alkaline hydrolysis with minimal NaOH consumption under unprecedently mild conditions. Complete PET depolymerization with 96% TPA yield is achieved at atmospheric pressure within 5 min at 90 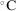, or in less than 2 h at 25 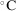. The method is here demonstrated using propylene carbonate (PC), one of the greenest,^[^ ^33^, ^34^ ^]^ non‐volatile, and most cost‐effective solvents available on the market. The strategy leverages the high solubility of PET in PC at elevated temperature to achieve a rapid complete dissolution, and PC's good water miscibility at ambient temperature to crucially enable full replacement of the retained solvent after thermally induced phase separation (TIPS). While preventing the re‐compaction of polymer chains into a hydrophobic solid, interfacial water facilitates depolymerization by significantly enhancing the transport of hydroxyl ions to the ester bonds, thereby minimizing NaOH consumption. The robustness of the strategy is demonstrated by high efficiency on challenging post‐consumer PET products—such as colored textiles and mixed feedstocks—without being hindered by PET crystallinity. Comparison with recent reports highlights the superiority of this approach, which achieves a more than 20‐fold reduction in environmental energy footprint compared to conventional direct PET hydrolysis, while addressing key scalability challenges associated with current alkaline methods. A techno‐economic analysis (TEA) provides quantitative assessment of the process feasibility under industrial‐scale conditions, highlighting energy efficiency and strong economic viability. Overall, this study introduces a novel concept in depolymerization strategy, based on polymer dissolution and TIPS followed by water replacement, with broad applicability beyond PET.

## Results and Discussion

### Obtaining Water‐Swollen PET

**Figure 1:**
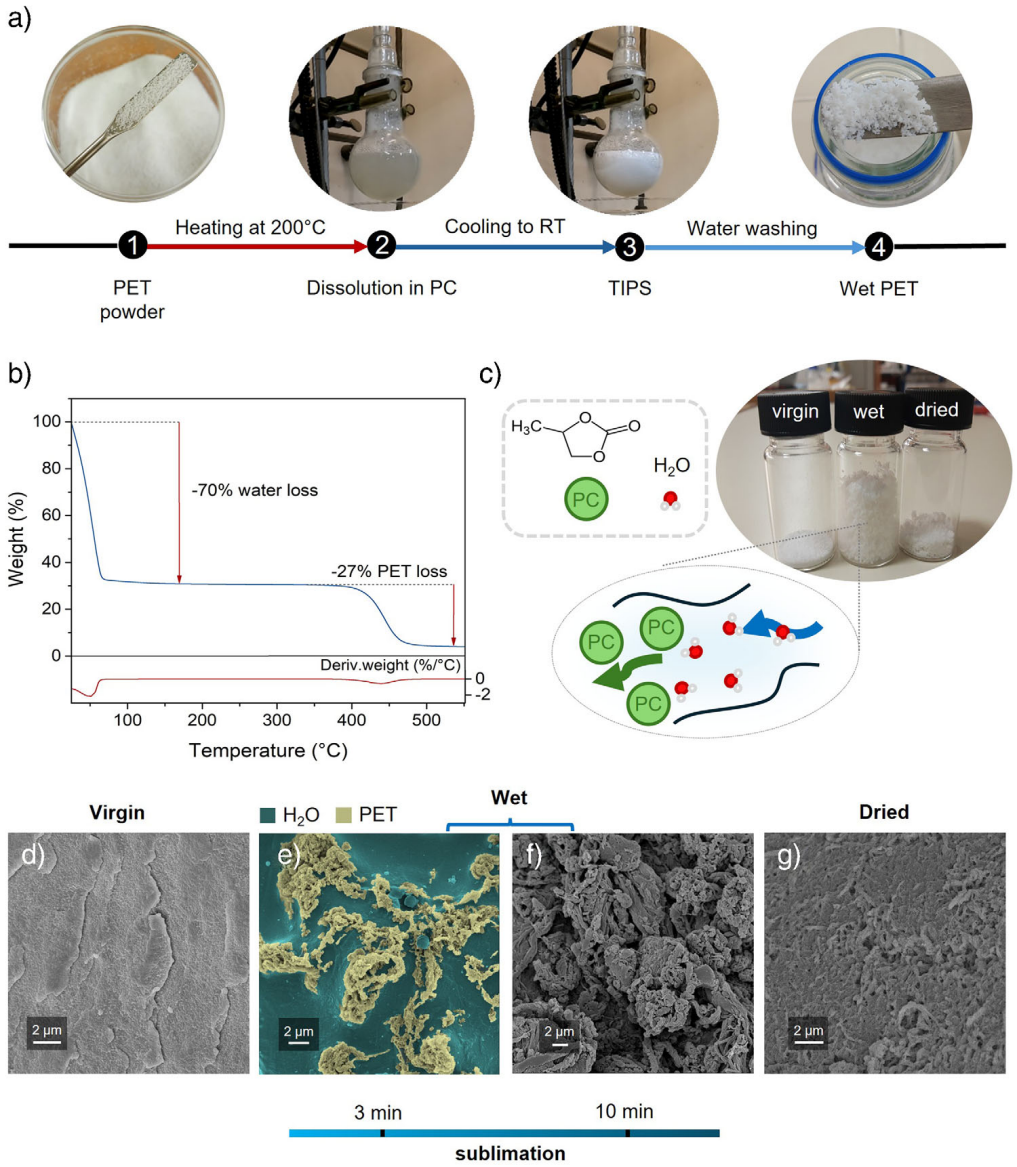
a) Procedure for preparing water‐swollen PET. b) TGA of water‐swollen PET. c) Volume comparison (1 g) of virgin, wet, and dried PET. d) SEM image of virgin PET. e),f) Cryo‐SEM images of the cryo‐fractured surface of wet PET after 3 min e) and 10 min f) of ice sublimation at ‐110 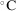. g) SEM image of dried PET.

PC was identified as a solvent capable of dissolving PET and delivering excellent performance throughout the entire process. Ranked among the greenest solvents according to the GSK sustainability guide^[^ ^33^, ^34^ ^]^ due to its low toxicity, high biodegradability, and cost‐effective production (Table S3), PC also features high polarity, high boiling‐point, low vapor pressure, and excellent thermal stability. A commercial PET powder with around 40% crystallinity was used in the experiments to model recalcitrant feedstocks. Although PET is insoluble in PC below 190 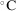 (Section S2.1), it readily dissolves at 200 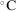 (over 40 wt%) and undergoes TIPS upon cooling. An optimal 25 wt% polymer loading ensures complete dissolution within 10 min, preventing excessive solution viscosity and minimizing polymer exposure to high temperature. Cooling to ambient temperature triggers phase separation leading to a sponge‐like porous solid that retains the solvent. Through washing, the aqueous solubility of PC (240 ${\mathrm{gL}}^{-1}$ at 20 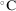) enables its gradual and complete replacement by water within the voids between polymer chains, while preventing their re‐compaction (Figure 1a). Water is used in a mass ratio of 5:1 relative to PC to ensure efficient washing. Despite the hydrophobic nature of PET (maximum water uptake ∼1% under saturated moisture conditions^[^ ^35^ ^]^), the polymer at this stage entraps an exceptional amount of water within its structure, thereby forming a stable water‐swollen PET phase. Thermogravimetric analysis (TGA) reveals an uptake of ∼230% (Figure 1b), accompanied by substantial volume expansion (Figure 1c). Upon oven‐drying overnight at 70 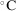, the sample undergoes significant volume contraction due to water loss, yet does not revert to its original compact structure. Scanning electron microscopy (SEM) and cryogenic‐SEM provided insights into the distinct morphological characteristics of virgin, water‐swollen, and dried PET. While the untreated material exhibits a continuous, compact surface (Figure 1d), wet PET appears to consist of filamentary aggregates surrounded by micrometer‐scale, water‐rich regions (Figure 1e). Removal of these water‐rich regions by ice sublimation reveals that the polymer structure consists of tangled and intertwined chain bundles (Figure 1f). In contrast, water removal through evaporation allows the polymer chains to reaggregate, leaving only a limited residual porosity (Figure 1g). The treatment results in negligible loss of both polymer and dissolution solvent: PET was recovered with a yield exceeding 98%, based on the mass ratio of the dried product to the initial material, while PC was recovered after water separation (Section S1.3) with 98.4% purity, according to gas chromatography–mass spectrometry (GC‐MS) determination (Figure S5).

### Comparison Between Virgin and Dried PET

**Figure 2:**
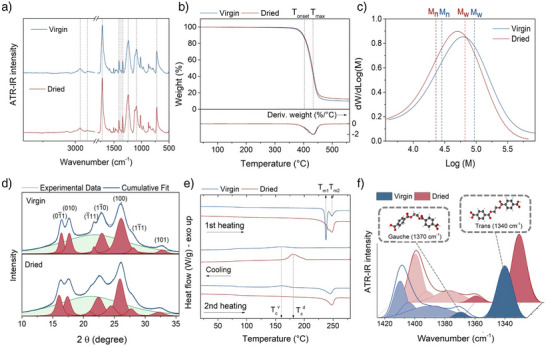
Comparison between virgin and dried PET. a) ATR‐IR spectra showing unchanged characteristic vibrations: C═O and ester stretching (1712, 1243, 1090 ${\mathrm{cm}}^{-1}$), aromatic ring modes (1410, 722 ${\mathrm{cm}}^{-1}$), and C–H stretching (2970, 2910 ${\mathrm{cm}}^{-1}$) and bending (1370, 1340 ${\mathrm{cm}}^{-1}$). b) TGA curves. c) GPC traces with indicated $M_{w}$ and $M_{n}$. d) WAXD patterns with multipeak fitting: red peaks correspond to crystalline reflections, green band to the amorphous halo. e) DSC curves (first heating, cooling, second heating; 10 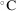 $\min^{-1}$): upward arrows indicate melting peaks of virgin PET ($T_{m1}$=237 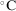, $T_{m2}$=247 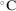); downward arrows indicate crystallization peaks upon cooling in virgin ($T_{c}^{v}$=159 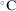) and dried ($T_{c}^{d}$=179 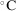) PET. f) Band decomposition of ATR‐IR spectra (1320–1425 ${\mathrm{cm}}^{-1}$) highlighting wagging modes of glycol segments in trans‐extended (1340 ${\mathrm{cm}}^{-1}$) and gauche‐twisted (1370 ${\mathrm{cm}}^{-1}$) conformations.

The polymer's stability during the formation of water‐swollen PET was evaluated by attenuated total reflection infrared (ATR‐IR) spectroscopy, TGA, gel permeation chromatography (GPC), wide angle x‐ray diffraction (WAXD), and differential scanning calorimetry (DSC). The ATR‐IR spectrum of the dried sample matches that of virgin PET (Figure 2a), indicating the preservation of functional groups. No OH‐stretching band of carboxylic and alcoholic chain end groups appears in the 3200–3500 ${\mathrm{cm}}^{-1}$ region, excluding severe polymer degradation.^[^ ^36^ ^]^ TGA further confirms structural integrity, with comparable degradation onset (*T* 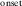) and maximum degradation rate (*T* 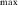) temperatures for virgin and dried samples (Figure 2b, Table S4). However, GPC reveals a clear shift toward lower molecular weights (Figure 2c, Table S5) due to hydrolytic chain scission by residual moisture, accompanied by a modest narrowing of the molecular weight distribution (PDI decreasing from 3.2 to 2.8). As indicated by 27% drop in *M* 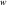 versus 17% in *M* 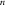, this narrowing is the result of statistically more probable cleavage of longer chains, consistent with random scission. Therefore, although PC‐induced dissolution compromises PET's mechanical integrity, making it unsuitable for direct reuse, it is advantageous as a preparatory step for efficient depolymerization.

WAXD and DSC analyses were used to assess the effect of the treatment on the crystal structure—a key factor in PET's degradation behavior.^[^ ^16^, ^37^ ^]^ Unlike lignocellulosic materials,^[^ ^38^ ^]^ no amorphization was observed: both virgin and dried PET retained a crystallinity of ∼40% (Figure 2d,e). However, differences emerged in crystal structure. WAXD shows smaller average crystallite size along the chain axis in dried PET (2.5 nm) versus virgin PET (3.5 nm) (Figure 2d, Table S6). Furthermore, DSC reveals that in the first heating scan virgin PET exhibits two distinct melting peaks—corresponding to crystals formed during primary and secondary crystallization^[^ ^39^ ^]^—whereas the dried sample displays two closely overlapping peaks, dominated by the high‐temperature component, resulting in a broadened signal that indicates a wider crystallite size distribution and greater recrystallization tendency. This is supported by the cooling scan, where treated PET crystallizes 20 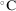 earlier and more sharply than virgin PET, and by the higher crystallinity after reheating (26% vs. 12%) (Figure 2e and Table S7). These changes arise from polymer chain disentanglement during dissolution, which facilitates an extended conformation upon precipitation and lowers the energy barrier for crystallization.^[^ ^40^, ^41^ ^]^ ATR‐IR analysis (Figure 2f) confirms this, with ∼77% of chains in the treated sample adopting a trans‐extended conformation, compared to ∼48% in the virgin polymer.

### Depolymerization by Alkaline Hydrolysis

**Figure 3:**
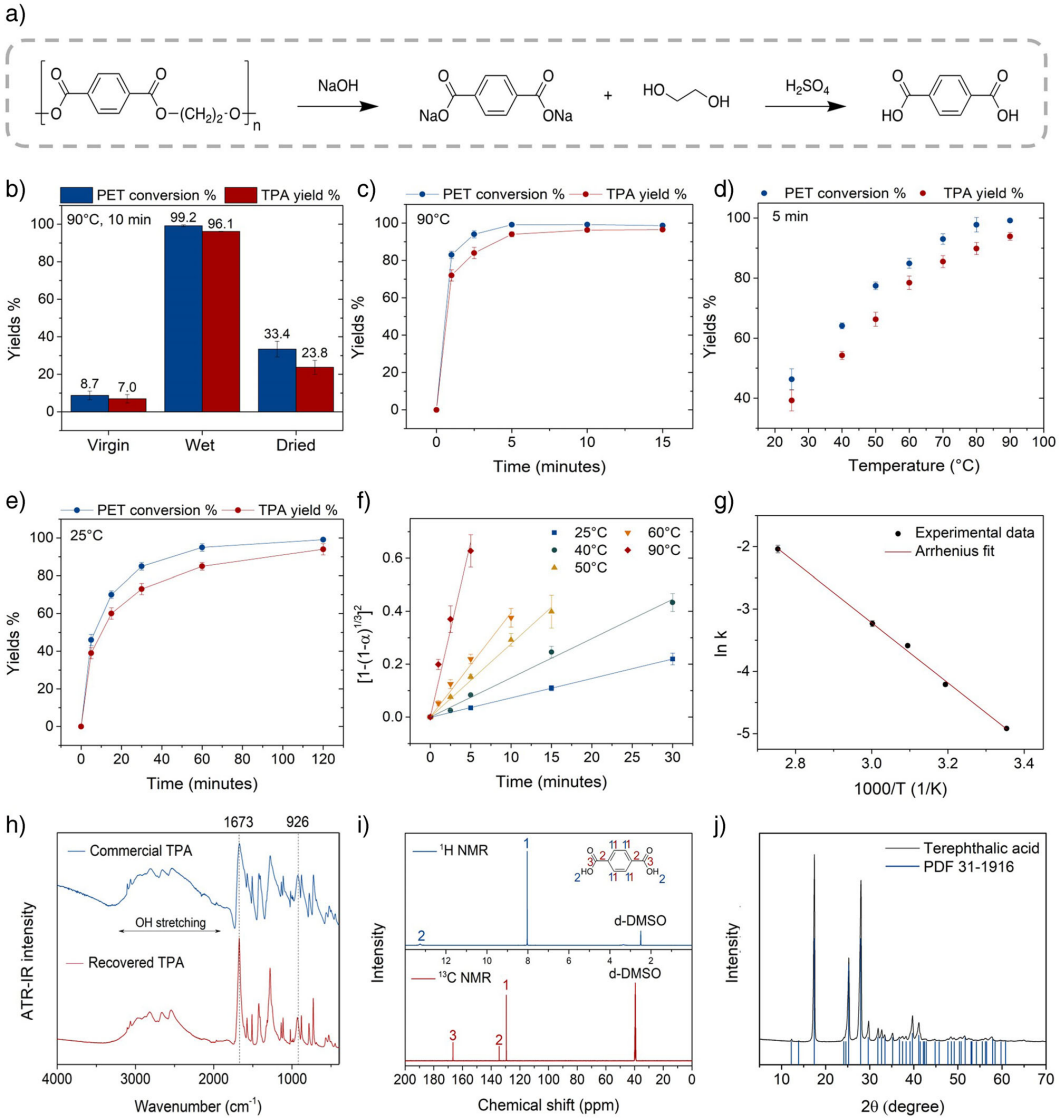
a) Reaction scheme for TPA recovery. b) PET conversion and TPA yield after 10 min hydrolysis at 90 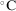 for virgin, wet, and dried PET. c) Time dependence of PET conversion and TPA yield during hydrolysis of wet PET at 90 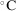. d) Temperature‐dependent PET conversion and TPA yield after 5 min hydrolysis at 90 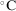 of wet PET. e) Time dependence of PET conversion and TPA yield during hydrolysis of wet PET at 25 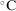. f) Jander plot of wet‐PET conversion (${\left[1-{\left(1-\alpha \right)}^{\frac{1}{3}}\right]}^{2}$ vs. t) at different temperatures; solid lines: Jander model fits. g) Arrhenius plot of rate constant (k) from Jander model; solid line: fit to $k=k_{0}\exp\left(-E_{a}/RT\right)$. h) ATR‐IR spectra of recovered TPA and commercial standard, highlighting carboxylic acid bands. i) 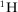 and 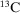 NMR spectra of recovered TPA. j) WAXD pattern of recovered TPA compared to reference (triclinic TPA, PDF 00‐031‐1916; blue bars). Error bars in b)—f): standard deviation of three independent replicates.

NaOH‐promoted hydrolysis converts PET into water‐soluble disodium terephthalate (${\mathrm{Na}}_{2}\mathrm{TPA}$) and ethylene glycol (EG), with TPA recovered through acidification with sulphuric acid ($H_{2}$ ${\mathrm{SO}}_{4}$) (Figure 3a). Compared to HCl, $H_{2}$ ${\mathrm{SO}}_{4}$ is less volatile, cheaper, and its consumption is lower. To evaluate the impact of PET pretreatment on depolymerization, virgin, water‐swollen, and dried samples were tested for 10 min under identical conditions (NaOH:PET = 0.5:1 w/w; added $H_{2}O$:PET = 3:1 w/w; 90 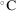; 0.1 MPa). The low NaOH amount—slightly above the stoichiometric minimum—minimizes acid consumption for TPA precipitation, while the high solid loading reduces wastewater generation. Notice that the 4.17 M NaOH concentration in the dry samples drops to 2.36 M for wet PET, due to the water content within the polymer matrix, thereby generating less corrosive conditions. As shown in Figure 3b, virgin PET undergoes minimal depolymerization (<9%) with only 7% TPA yield, whereas the water‐swollen sample achieves complete depolymerization with a 96% yield. Dried PET reaches just 33% conversion and 23% yield. Although full hydrolysis of dried PET occurs within ∼3 h—markedly faster than the 16 h required for untreated PET (Table S8, entry 4)—its performance remains significantly inferior to that of the water‐swollen counterpart and unacceptably low for commercial application. These results underscore the benefit of residual porosity, reduced crystallite size, and chain disentanglement; however, the dramatic acceleration observed for the wet sample demonstrates the critical role of overcoming PET's hydrophobicity, which enables nucleophilic attack on a scale beyond the reach of existing methods.

Encouraged by these results, we investigated the hydrolysis kinetics of water‐swollen PET in detail. At 90 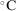, the reaction proceeds extraordinarily fast: 80% conversion is reached in just 1 min, and complete depolymerization is achieved within 5 min (Figure 3c), corresponding to a ∼200‐fold acceleration compared to virgin PET. Remarkably, high depolymerization rates are maintained even at significantly lower temperatures. As shown in Figure 3d,e, PET conversion and TPA yield at 5 min gradually decrease with temperature, yet complete hydrolysis is still achieved at room temperature (25 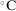) within ∼2 h. To our knowledge, this is the first report of complete hydrolysis of PET in water at ambient temperature and pressure using a near‐stoichiometric amount of NaOH. Furthermore, full depolymerization without external heating is attained in just 15 min by doubling the amount of NaOH, leveraging the exothermic dissolution of the hydroxide (Table S9, entry 31). However, this faster route increases acid consumption for TPA precipitation, making it less favorable in terms of atom economy.

Figure 3f shows that the hydrolysis kinetics at different temperatures is well described by the Jander equation, ${\left[1-{\left(1-\alpha \right)}^{\frac{1}{3}}\right]}^{2}=kt$, where α is the PET conversion at time t and k is the rate constant.^[^ ^42^ ^]^ Commonly applied to diffusion‐controlled processes in solid‐state chemistry,^[^ ^43^ ^]^ this model provides a reasonable fit to the experimental data, consistent with the slurry‐like nature of the reaction mixture under high solid loading. The temperature dependence of k follows an Arrhenius behavior (Figure 3g), yielding an activation energy (*E* 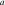) of 40.1±0.4 kJ ${\mathrm{mol}}^{-1}$. This value is substantially lower than those typically reported for PET hydrolysis (60–80 kJ ${\mathrm{mol}}^{-1}$),^[^ ^22^, ^44^, ^45^, ^46^ ^]^ further highlighting the critical role of pretreatment in markedly accelerating the reaction.

The recovered TPA was characterized by ATR‐IR spectroscopy, 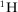 and 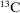 NMR, and WAXD. ATR‐IR analysis (Figure 3h) confirms a spectral profile identical to that of a commercial TPA standard, featuring the typical carboxylic acid bands: broad O–H stretching (2200–3300 ${\mathrm{cm}}^{-1}$), C═O stretching (∼1670 ${\mathrm{cm}}^{-1}$), and O─H rocking (∼920 ${\mathrm{cm}}^{-1}$).^[^ ^29^ ^]^ The 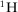 NMR spectrum shows only two resonances—at 13.25 ppm (hydroxyl protons) and 8.03 ppm (aromatic protons)—consistent with the expected TPA structure. The 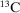 NMR spectrum shows three peaks at 166.62, 134.43, and 129.41 ppm, corresponding to the carbonyl, quaternary aromatic, and aromatic carbons, respectively,^[^ ^8^ ^]^ with no additional signals, indicating negligible impurities (Figure 3i). WAXD analysis (Figure 3j) confirms the crystalline nature of the product, matching the reference pattern for triclinic TPA (PDF 00‐031‐1916). Together, these results demonstrate that the method successfully recovers highly pure, crystalline TPA.

Comparable PET conversion and TPA yield were achieved using ethylene carbonate (EC)—a low‐cost solvent with broad industrial use—as an alternative to PC for polymer dissolution (Table S8, entry 6). Although EC is less favorable from a sustainability standpoint due to higher health concerns,^[^ ^34^ ^]^ this water–miscible, high‐boiling solvent with a high dipole moment still enables the physical mechanism underpinning ultrafast hydrolysis, i.e., polymer dissolution and TIPS, followed by water replacement. These findings highlight the versatility of the approach and provide a basis for expanding the solvent scope.

### Processing of Post‐Consumer and Mixed Waste

**Figure 4:**
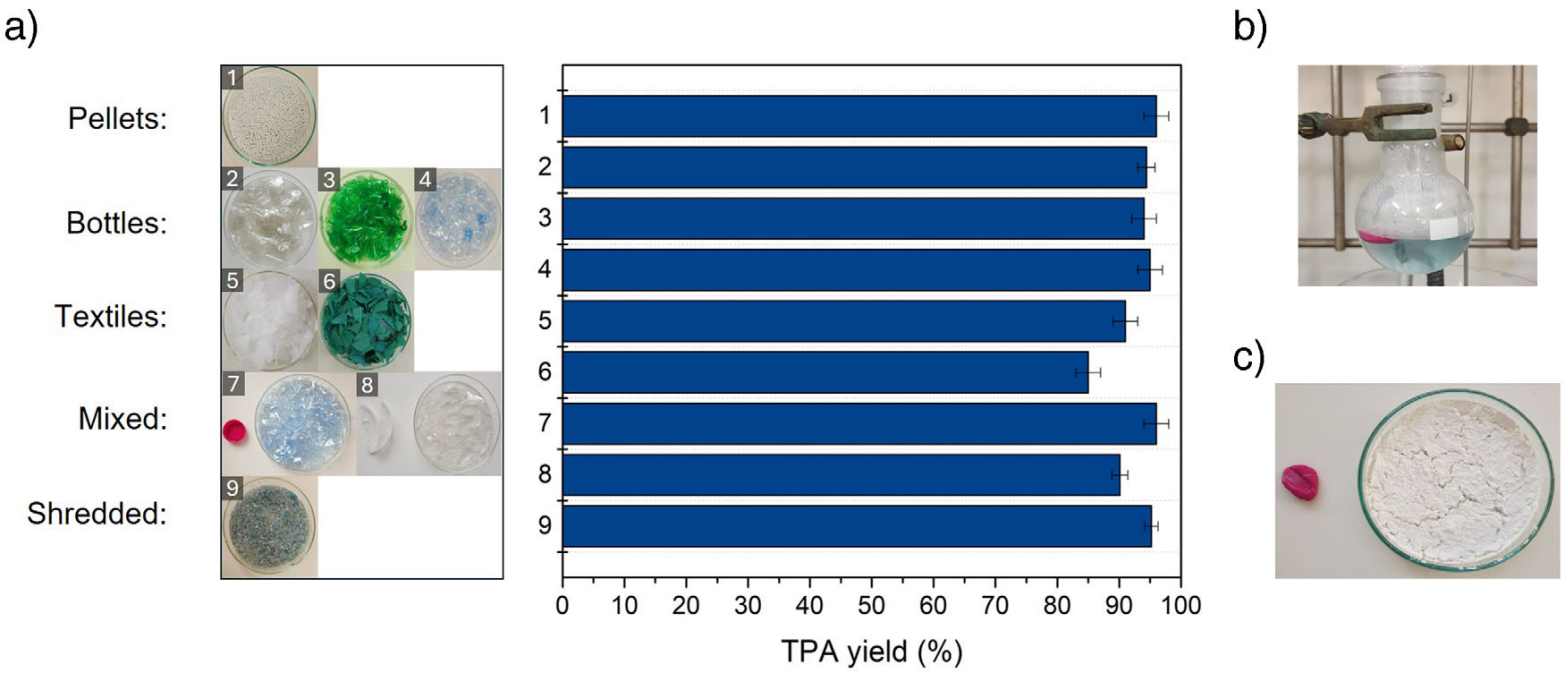
a) Photographs of PET‐containing samples: pellets (1), transparent/colored bottles (2–4), textiles (5,6), mixed plastics (7,8), and shredded mixed waste (9). Bar graph shows TPA yield after pretreatment in PC at 200 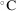 and alkaline hydrolysis at 90 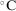 for 10 min (NaOH:PET=0.5 w/w). Error bars represent standard deviation of three independent replicates. b) Selective dissolution of a blue PET bottle with PE cap (sample 7) in PC at 200 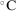; the PE cap remains undissolved and buoyant. c) PE cap recovered post‐treatment and TPA isolated after hydrolysis of the PET bottle.

The accelerated hydrolysis of water‐swollen PET was demonstrated across a wide range of pre‐ and post‐consumer products, including virgin pellets, colored bottles, textiles, and shredded PET waste from a recycling facility (Figure 4a). The latter provides a realistic benchmark for evaluating the process's robustness against contaminated and degraded feedstocks typical of commercial recycling streams. Dissolution in PC at 200 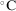 was complete within 10 min for bottles and textiles, and within 25 min for pellets (Tables S10 and S11). PC selectively dissolves PET, enabling straightforward separation of insoluble polyolefins (e.g., PE, PP), which float on the solvent surface and can be removed by tweezers or filtration (Figure 4b). Following 10 min of alkaline hydrolysis at 90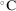, TPA yield exceeded 94% for bottles and reached 85% for textiles—particularly noteworthy given the recalcitrant nature of PET fibers.^[^ ^8^, ^47^ ^]^ For shredded waste, a minor insoluble fraction remained after 25 min, likely due to contaminants reducing PC's solvating power. However, hydrolyzing both the soluble and insoluble fractions still produced 95% TPA, indicating that incomplete dissolution does not impair hydrolysis efficiency. In constrast, 10 min of hydrolysis at 90 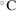 are completely ineffective without pretreatment (Tables S10 and S11). All treated materials yielded high‐purity TPA with a bright white appearance (Figure 4c) and no detectable oligomers, as confirmed by ATR‐IR (Figure S6). Only green‐colored samples retained a faint coloration (Table S10), which was fully removed via a simple post‐treatment with activated carbon (Figure S7).

### Sustainability Assessments

**Figure 5:**
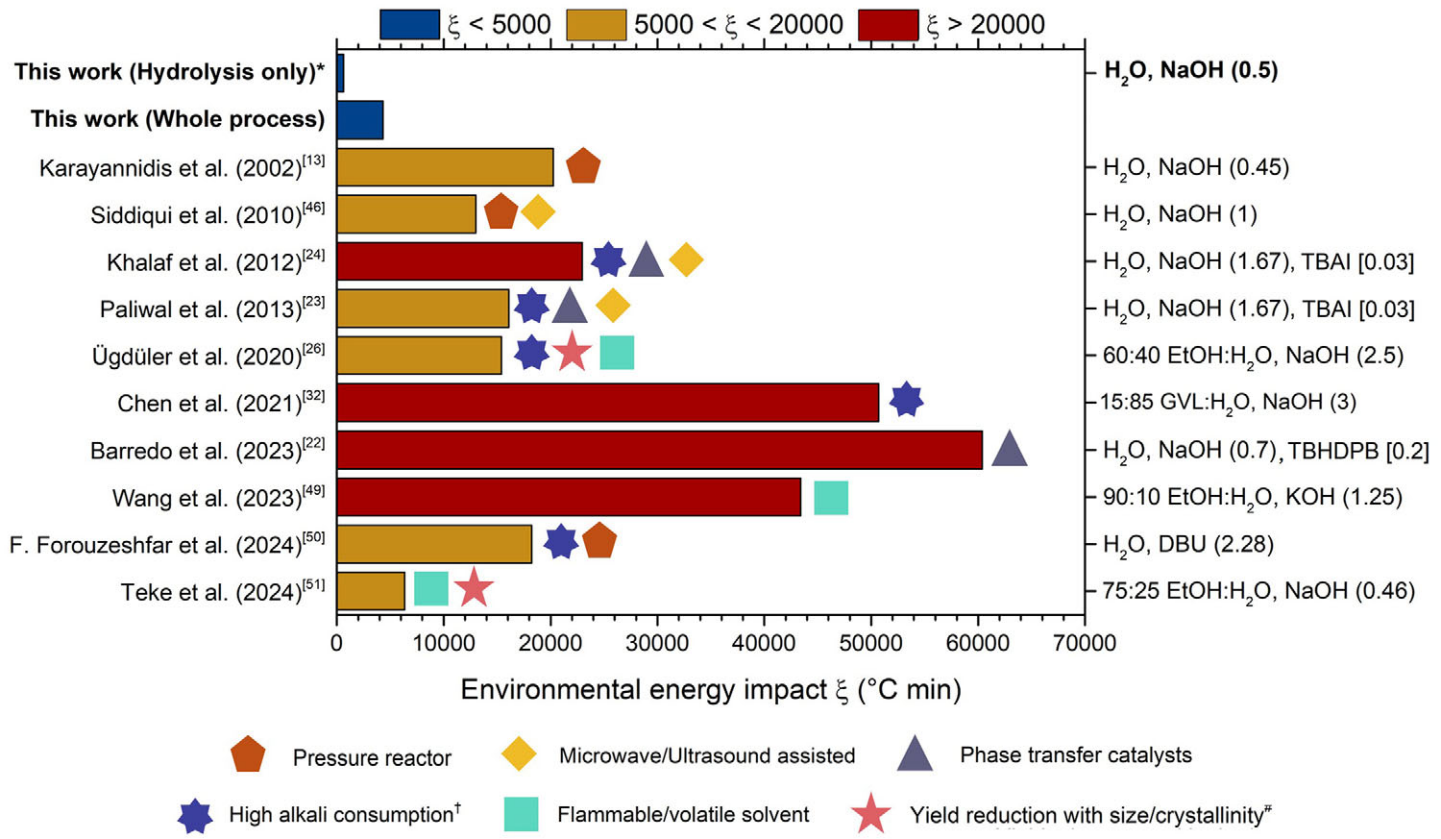
Comparison of the environmental energy impact factor (ξ) for various aqueous alkaline hydrolysis methods. Left axis: literature references. Right axis: solvent composition (vol %), alkaline reagent, alkali‐to‐PET mass ratio (round brackets), catalyst, catalyst‐to‐PET mass ratio (square brackets). Key limitations are indicated by geometric symbols (see legend). 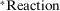 conditions: PET (1 g), $H_{2}O$ (3 mL), NaOH (0.5 g), at 90 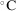 for 5 min. 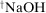:PET>1:1 (w/w). 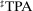 yield <50%.

To assess the sustainability of the process, we applied the green chemistry metrics parameters proposed by Barnard et al.,^[^ ^48^ ^]^ adapted to include the PET's pretreatment (Section S2.2). The environmental energy impact factor (ξ) served as a key benchmark, with lower values indicating higher sustainability. A remarkably low value of 4325  C·min—nearly 20 times lower than that of virgin PET (88794  C·min)—establishes 5 min at 90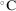 as the optimal condition for complete hydrolysis (Figure S8). Notably, 86% of this energy impact stems from the high‐temperature pretreatment, whereas the alkaline hydrolysis itself contributes only a small fraction (14%), underscoring its intrinsic energy efficiency. Figure 5 compares our process with other alkaline hydrolysis methods in aqueous solvent reported in the literature.^[^ ^13^, ^22^, ^23^, ^24^, ^26^, ^32^, ^46^, ^49^, ^50^, ^51^ ^]^ The process stands out as the most sustainable and industrially scalable, offering several key advantages. First, the use of PC—a high‐boiling, non‐volatile solvent—for PET dissolution, in combination with mild hydrolysis conditions, eliminates the need for costly high‐pressure or microwave reactors, which remain major barriers to industrial deployment. Notably, the synthesis of PC using ${\mathrm{CO}}_{2}$ captured from industrial emissions (e.g., fermentation, combustion, or chemical plants)^[^ ^52^ ^]^ effectively couples carbon dioxide valorization with the production of a green solvent (non‐toxic, non‐flammable, and biodegradable). Second, the exclusion of hazardous solvents such as dichloromethane and methanol significantly enhances safety and reduces regulatory constraints. Third, the low alkali usage reduces reagent consumption and minimizes downstream costs associated with neutralization and salt byproduct management. Notably, ${\mathrm{Na}}_{2}$ ${\mathrm{SO}}_{4}$ is not a process waste but offers additional revenue potential. Moreover, the NaOH concentration used (2.36 M) is fully compatible with industrial‐scale operations, supporting straightforward scalability. Finally, the high depolymerization efficiency on crystalline PET enables the processing of recalcitrant real‐world feedstocks, further underscoring the potential for industrial viability of the method.

### Techno‐Economic Analysis

**Figure 6:**
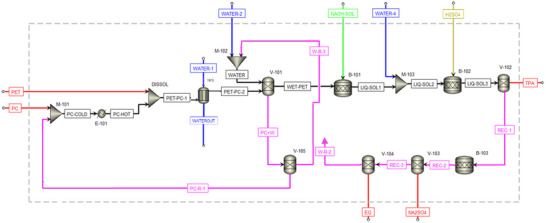
Process flowsheet of the PET recycling plant simulated in Aspen Plus.

To evaluate TPA production at the industrial scale, we conducted a techno‐economic analysis (TEA) in Aspen Plus for a recycling plant processing PET at a throughput of 1 ton $h^{-1}$. The analysis was based on the experimental laboratory process, adapted to industrial conditions and incorporating optimization and material recycling to minimize waste in line with a circular‐economy approach (Figure S2). The process simulation flowsheet (Figure 6) includes all equipment and process streams, illustrating how the input materials (PET, PC, water, $H_{2}$ ${\mathrm{SO}}_{4}$, and NaOH) are converted through the various steps to yield TPA, together with secondary products (${\mathrm{Na}}_{2}$ ${\mathrm{SO}}_{4}$ and EG) and purge streams that prevent material accumulation in the system.

Mass and energy balances obtained from the simulation were integrated into cost models to estimate operating expenditures (Table S2, Figures S3 and S4). The results indicate a total operating cost of US$612 ${\mathrm{ton}}^{-1}$ PET, in line with market benchmarks,^[^ ^53^, ^54^, ^55^ ^]^ and a total energy consumption of US$36.18 ${\mathrm{ton}}^{-1}$ PET, competitive with existing technologies. To ensure a consistent techno‐economic comparison (Table S12), the energy cost data from the literature were adjusted to current market energy prices.

A closer examination of the energy and cost distribution reveals that heating and dissolving PET in PC at 200 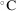
—a key step in the process—contributes about 44% of the total plant operating cost and accounts for about 61% of the total energy consumption. Although PET pretreatment in PC represents the most cost‐intensive step, it enables high overall energy efficiency by allowing the subsequent steps to operate under significantly milder and less demanding conditions. Notably, valorization and recycling of all solid and liquid streams within the process boundary—including full reuse of the aqueous phase—enable a zero‐waste and zero‐liquid‐discharge operation, underscoring the circular and sustainable nature of the proposed strategy.

## Conclusion

Recycling PET through chemical yet sustainable routes is a urgent need to ensure the continued use of this plastic while preserving the environment and preventing global resources depletion. This study introduces a strategy to entrap exceptional amounts of water within the hydrophobic polymer, enabling ultrafast alkaline hydrolysis under unprecedently mild conditions with minimal alkali consumption. The method, for the first time, has been demonstrated using PC as PET dissolution agent. The solvent initially remains intercalated within the interconnected channels of the porous polymer matrix formed by TIPS and then, owing to its water solubility, acts as a carrier to introduce water between the polymer chains, eventually being fully replaced. Interfacial water stabilizes chain separation and establishes a highly effective environment for hydroxyl ion transport, which profoundly accelerates depolymerization (complete hydrolysis at atmospheric pressure in 5 min at 90 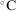 or even in less than 2 h at room temperature, with 96% TPA yield). The method proves remarkably effective on challenging and recalcitrant feedstocks, including colored textiles and mixed plastic waste, enabling selective depolymerization without being hindered by PET crystallinity. Green chemistry metrics reveal a 20‐fold reduction in environmental energy impact compared to direct PET hydrolysis, while comparison with recent alkaline hydrolysis methods highlights the overcoming of key limitations that hinder the sustainable scalability of current methods. TEA further validates the technical feasibility of the process under industrially relevant conditions (1 ton $h^{-1}$ PET). With an energy demand of US$36.18 ${\mathrm{ton}}^{-1}$ PET and an operating cost of US$612 ${\mathrm{ton}}^{-1}$ PET, the process demonstrates favorable energy efficiency and overall competitiveness compared to existing engineered technologies. Moreover, optimization and recycling steps enable nearly‐zero waste and position the process within a circular economy.

The mechanistic foundation of this new strategy ensures broad versatility, enabling its application to other condensation polymers beyond PET and supporting the use of dissolution solvents beyond PC. On one hand, PC demonstrates dissolution capability toward other polyesters (e.g., PBT, PLA), polycarbonates (e.g., BPA‐PC), and polyamides (e.g., PA6, PA66), as confirmed by in‐house laboratory tests. On the other hand, while PC offers an optimal balance between chemical performance and adherence to green chemistry principles, other cyclic carbonates (e.g., EC) also hold promise, as they may enable the same physical mechanism that drives the exceptional rate enhancement of hydrolysis—i.e., polymer dissolution and TIPS, followed by water replacement—while maintaining a favorable environmental sustainability profile. Altogether, the novel approach demonstrates a rare combination of high chemical efficiency, low environmental burden, and strong potential for scale‐up, significantly advancing the prospects for sustainable recycling of a broad range of plastic waste. Reflecting its practical relevance, it is the subject of both a national (Italian) and a corresponding international patent application.

## Author Contributions

F.M., R.G., and S.C. conceived the project. S.C. supervised the work and acquired funding. F.M. performed the experiments, analyzed the data, and provided interpretation. L.C. conducted the techno‐economic analysis (TEA). P.S., A.D., and M.C. contributed to IR, WAXD, and cryo‐SEM measurements, respectively. S.G. provided resources. F.M. wrote the original draft, and F.M. and S.C. finalized the manuscript. All authors contributed to data interpretation, read, and approved the final version of the manuscript.

## Conflict of Interests

The authors declare no conflict of interest.

## Supporting information

Supporting Information

## Data Availability

The data that support the findings of this study are available in the Supporting Information of this article.
