# Supplementary material for: Overcoming Hydrophobicity with Water Enables Ultrafast Hydrolysis of Waste Polyethylene Terephthalate at Very Mild Conditions
Source: Angew Chem Int Ed Engl. 2025 Nov 10;65(1):e14136. doi: 10.1002/anie.202514136 (PMC12759205; doi:10.1002/anie.202514136)
Supplement: Supplementary file 1 — Supporting Information [file ANIE-65-e14136-s001.pdf]

Supporting Information

Overcoming Hydrophobicity with Water Enables Ultrafast Hydrolysis of Waste Polyethylene Terephthalate at Very Mild Conditions

Francesco Millucci[a], Raimondo Germani[b,*], Leonardo Colelli[c], Serena Gabrielli[d], Paola Sassi[b], Anna Donnadio[e], Martina Conti[f], Silvia Corezzi[a,*]

[a] *Dipartimento di Fisica e Geologia, Università di Perugia, I-06123 Perugia, Italy*
[b] *Dipartimento di Chimica, Biologia e Biotecnologie, Università di Perugia, I-06123 Perugia, Italy*
[c] *Department of Chemical Engineering Materials Environment, "Sapienza" University of Rome, I-00184 Rome, Italy*
[d] *Chemistry Interdisciplinary Project (ChIP), Scuola di Scienze e Tecnologie, Università di Camerino, I-62032 Camerino, Italy*
[e] *Dipartimento di Scienze Farmaceutiche, Università di Perugia, I-06123 Perugia, Italy*
[f] *CNR - Istituto Officina dei Materiali (IOM), Area Science Park Basovizza, I-34149 Trieste, Italy*

[*] *Corresponding authors: silvia.corezzi@unipg.it; raimondo.germani@unipg.it*

1 Experimental Section

S1.1 Materials

Polyethylene terephthalate (PET) powder (sieved at 1200 μm: max particle size 1600 μm; average particle size 500 μm) and pellets (cubical shape; size 2.5 mm) were provided by Poliplast S.r.l., Italy. Post-consumer PET products, including water bottles, textiles, and containers, were obtained from local retailers, washed, dried, and cut into ~1 cm × 1 cm pieces before depolymerization experiments. Shredded PET waste was collected from a local recycling plant. Sodium hydroxyde (NaOH) (M_w=39.9971, by Merck), sulphuric acid (H_2SO_4) (M_w=98.09, by Sigma-Aldrich), terephthalic acid (TPA) (M_w=166.14, by Carlo Erba), propylene carbonate (PC) (M_w=102.089, by Seven B-Oil), and ethylene carbonate (EC) (M_w=88.062, by Seven B-Oil) were all purchased with >99% purity and used as received. Water content in PC, determined by Karl Fischer titration (Metrohm 684 KF Coulometer), was less than 1 wt%.

S1.2 PET pretreatment with PC

In a typical pretreatment, 60 g of PC were weighed and transferred into a 100 mL two-necked round-bottom flask equipped with a mechanical stirrer. The flask was then immersed in a silicone oil bath preheated to 200 °C. Next, 20 g of PET powder, previously dried overnight at 70 °C in oven, were added to the hot solvent and continuously stirred at 250 rpm for 10 min, ensuring complete dissolution. The flask was then removed from the silicone bath, and its content was quickly poured into a glass crystallization bowl, where rapid cooling-induced phase separation occurred. The resulting material was powdered using a blender, thoroughly washed with distilled ultra-pure deionized water until complete removal of PC, and stored into a sealed glass bottle at room temperature for use within 2–3 weeks. During the washing step, water was employed in a quantity corresponding to five times the mass of PC. The same procedure was applied using EC as an alternative solvent to PC. For post-consumer products, the dissolution time in PC was extended as necessary to achieve complete dissolution.

S1.3 Recovery of PC

PC (b.p. 242 °C) does not form an azeotrope with water (b.p. 100 °C); therefore, simple distillation allowed efficient recovery of PC. In particular, water in PC (mass ratio 5:1) was readily removed by vacuum distillation (15 mmHg, membrane pump) at 50 °C.

S1.4 Alkaline hydrolysis of PET

We performed alkaline hydrolysis experiments on PET samples of 1 or 5 g. The procedure is described here for 1 g of PET. The sample was weighed and transferred into a 50 mL flask equipped with a mechanical stirrer, then placed in an oil bath preheated to the desired reaction temperature. For experiments on wet PET, the amount of material containing 1 g of PET was determined from a sample dried in an oven at 70 °C overnight before conducting the experiment. Then, 0.5 g of NaOH was dissolved in 3 mL of water and added to the flask to initiate the reaction. Under these conditions, the mixture formed a slurry, which was stirred at 250 rpm throughout the reaction. We conducted reactions of different durations at various temperatures (25, 40, 50, 60, and 90 °C). At the end of each reaction, the mixture was rapidly cooled by immersing the flask in ice water, and

50 mL of distilled water was added to dissolve the generated products. Unreacted PET fragments and insoluble oligomers were separated by filtration, rinsed with distilled water, dried in an oven, and then weighed. The filtrate was acidified with H_2SO_4 to adjust the pH to approximately 2.5–3, inducing TPA precipitation. The precipitate was isolated by filtration, thoroughly rinsed with distilled water, dried in an oven, and weighed. PET conversion and TPA yield were calculated as:

$$PET\ conversion\,(\%) = \frac{reacted\ PET(g)}{starting\ PET\ (g)} \times 100 = \frac{starting\ PET\ (g) - residual\ PET\ (g)}{starting\ PET\ (g)} \times 100$$

$$TPA\ yield\,(\%) = \frac{TPA\ product\ (g)}{theoretical\ TPA\ yield\ (g)} \times 100 = \frac{TPA\ product\ (g)}{\frac{starting\ PET\ (g)}{192.16} \cdot 166.13} \times 100$$

where 192.16 and 166.13 are the molecular weight, respectively, of PET repeating unit and TPA.

S1.5 Decolorization of TPA

1 g of wet PET derived from green-colored bottles or textiles was subjected to alkaline hydrolysis at 90 °C for 10 min in the presence of 0.5 g of NaOH and 3 mL of water. At the end of the reaction, 20 mL of distilled water and activated carbon (0.2 g for green bottles and 0.5–1 g for green textiles) were added to the reaction flask, and the mixture was stirred for an additional 20 min. Unreacted PET and activated carbon were removed by filtration. The filtrate was then acidified with H_2SO_4 to precipitate TPA, which was isolated by filtration, thoroughly rinsed with distilled water, and dried in an oven.

S1.6 Characterizations

S1.6.1 Scanning electron microscopy

Scanning electron microscopy (SEM) images were obtained with a FE-SEM LEO 1525 ZEISS equipped with an Inlens detector and an angle-selective backscattered detector. Samples were mounted onto stubs and coated with a thin chromium layer before imaging. Cryogenic scanning electron microscopy (Cryo-SEM) images were obtained with a Leo Crossbeam 1540 XBFIB-SEM ZEISS. The sample was prepared by compressing a small amount of water-swollen PET powder between two metallic rivets loaded onto a custom-made ZEISS shuttle, and rapidly frozen by plunging into liquid nitrogen. The frozen shuttle was then transferred to the preparation chamber of a Quorum PP3010Z cryo stage maintained at a constant temperature of -140 °C. A pre-cooled knife was used to knocks off the top rivet and create a fracture surface. The sample was subjected to sublimation at -110 °C for 3 or 10 min, and then platinum sputtered for 60 s at 10 mA before imaging.

S1.6.2 Gas chromatography-mass spectrometry

An aliquot of PC recovered after water separation was diluted to 1000 ppm in chloroform ($CHCl_3$) and analyzed by gas chromatography-mass spectrometry (GC-MS) using a GC Agilent 8890 coupled with an Agilent 5977B/MSD system equipped with an Agilent DB-WAX (60 m x 0.25mm

x 0.25μm) column. The instrumental operating conditions were: injection temperature, 280 °C; helium flow rate, 1.2 mL/min (split 20:1); initial temperature, 160 °C (hold 1 min); heating rate, 15 °C/min to 250 °C (hold 1 min); MSD transfer line, 260 °C; MS source, 230 °C; MS quadrupole, 150 °C; MS in scan mode (29–500 amu).

S1.6.3 Attenuated total reflection infrared (ATR-IR) spectroscopy

ATR-IR spectra were acquired with a Bruker Alpha spectrometer equipped with an ATR module using a diamond crystal. Each spectrum was recorded with a resolution of 2 cm^{-1} across the wavenumber range of 300–4000 cm^{-1}, by averaging 30 scans. A sample press ensured close contact between the sample and the ATR crystal surface. For each acquisition, a background spectrum was collected using the clean ATR crystal. All spectra were baseline-corrected and normalized to the aromatic skeleton stretching band at $\sim$1410 cm^{-1}. Band decomposition in the spectral region 1320–1430 cm^{-1} was performed using five Gaussian peaks, and the fraction of glycol moieties in the trans conformation was estimated as:

$$T(\%) = \frac{A_{1340}}{A_{1340} + 6.7 \cdot A_{1370}} \tag{1}$$

where A_{1340} and A_{1370} are the areas of the peaks associated with the glycol wagging vibration in trans and gauche conformations, respectively, and $\epsilon_{1370}/\epsilon_{1340} = 6.7$ is the ratio between the corresponding absorption coefficients [1].

S1.6.4 Thermogravimetric analysis (TGA) and differential scanning calorimetry (DSC)

TGA was performed using a Netzsch STA 2500 Regulus thermal analyzer equipped with Al_2O_3 crucibles. A 10 mg sample was heated from room temperature to 800 °C under a nitrogen atmosphere at a heating rate of 5 °C/min.
DSC analyses were performed using a DSC 250 TA Instruments, with samples (2-3 mg each) sealed in Aluminium pans. Measurements were conducted under a nitrogen atmosphere at a rate of 10 °C/min, following a three-step thermal cycle: (I) first heating from 20 °C to 280 °C with a 2-min isothermal hold, (II) cooling to 20 °C with a 2-min isothermal hold, and (III) second heating to 280 °C. The degree of crystallinity of PET was calculated from the first heating scan using the equation: $X_c = (\Delta H_m - \Delta H_{cc})/\Delta H_m^0$, where ΔH_m is the melting enthalpy, ΔH_{cc} is the cold crystallization enthalpy, and ΔH_m^0 is the melting enthalpy of 100% crystalline PET (140.1 J/g).

S1.6.5 Gel permeation chromatography (GPC)

The molecular weight of PET samples was determined by GPC using an Agilent 1260 Infinity II Multi Detector Suite (MDS) equipped with three detectors: a dual-angle light scattering detector (15° and 90°), a refractive index (RI) detector, and a viscometer (VS) detector. Hexafluoroisopropanol with 20 mM potassium trifluoroacetate was used as the mobile phase at a flow rate of 0.03 mL/min. Data acquisition and analysis were performed using Agilent GPC/SEC software. The GPC system included an Agilent PL HFIPgel guard column and a PL HFIPgel (250 mm x 4.6 mm) column. Column calibration was performed using PMMA standards with M_p values ranging from 1010 to 1591000 g/mol.

S1.6.6 Nuclear magnetic resonance (NMR)

^{1}H and ^{13}C NMR spectra were acquired using a Bruker Ascend 500 Avance III HD spectrometer. Prior to analysis, TPA samples were dissolved in DMSO-d_6 and transferred into NMR tubes. ^{1}H and ^{13}C NMR data are reported as chemical shifts (ppm) referenced to the residual solvent peaks of DMSO-d_6 (2.50 ppm for ^{1}H and 39.6 ppm for ^{13}C).

S1.6.7 Wide angle x-ray diffraction (WAXD)

WAXD measurements were performed using a Bruker D8 Advance diffractometer in Bragg–Brentano geometry, equipped with a Lynxeye XE-T fast detector using the Cu-Kα radiation. The long fine focus (LFF) tube operated at 40 kV and 40 mA. Phase identification was conducted using Bruker DIFFRAC.EVA V5 and the PDF database. To minimize preferential orientation of microcrystals, samples were prepared as finely ground powder on a zero-background sample holder. Data were collected in the 2θ range of 5–70° with a step size of 0.017° and a counting time of 200 s per step. Diffraction patterns were analyzed using Philips Profit software, modeling both amorphous and crystalline peaks with pseudo-Voigt functions in the 10–35° 2θ range, following the protocol by Wang et al. [2]. The crystallite size (D) along the 001 chain direction was estimated from the crystallite size along the $0\bar{1}1$ reflection, as:

$$D_{001} = D_{0\bar{1}1} cos(\alpha) \tag{2}$$

where α, the angle between the 001 and $0\bar{1}1$ planes, is 67.7°.

2 Supplementary Notes

S2.1 PET dissolution in PC

Based on their structure, the molecular-level interactions between PC and PET are dominated by dipole-dipole coupling between the carbonyl groups. This is supported by ATR-IR spectroscopy of pure PC and of PC confined within the PET matrix after dissolution and TIPS (Fig. S1). Notably, the PET–PC interaction induces a blue shift of the carbonyl stretching band from 1780 to 1786 cm^{-1} [3], reflecting stronger dipolar interactions between PC molecules than between PET and PC. This suggests that dissolution is not enthalpically favored but is driven by the entropic contribution ($-T\Delta S$), consistent with the requirement of a sufficiently high temperature (∼200 °C). These findings are also consistent with Hansen solubility theory, which predicts a RED parameter of 2.48 for PET in PC at 25 °C, significantly above the threshold of 1 for favorable solvation (Table S1) [4, 5].

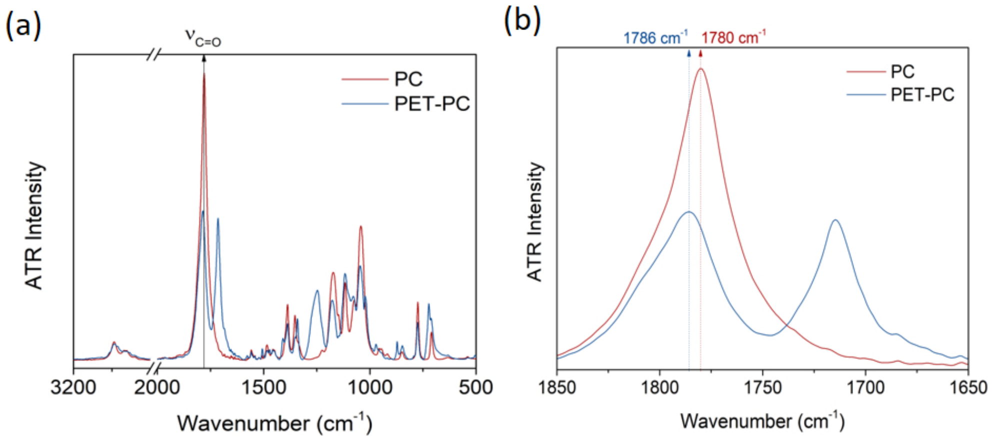

Figure S1: ATR-IR spectra of pure PC and of PC confined within the PET matrix after dissolution and TIPS, **(a)** recorded in the extended range 3200–500 cm^{-1}, and **(b)** in the frequency region 1850–1650 cm^{-1} of the PC carbonyl symmetric stretching vibration $\nu_{C=O}$ (marked by a dashed arrow).

Table S1: Hansen solubility parameters for PET and PC at $T = 25$ °C. RED_{PET} was calculated using an interaction radius R_0=5.0 $MPa^{1/2}$.

**Polymer/solvent**	**Hansen Solubility Parameters [$MPa^{1/2}$]**			**RED_{PET}**
	δ_D	δ_P	δ_H	
PET	18.2	6.4	6.6	
Propylene carbonate	20.0	18.0	4.1	2.48

S2.2 Calculation of green chemistry metrics

The green chemistry metrics recently proposed by Barnard et al. [6] were adapted to include the PET's water-swelling pretreatment, and employed for a comprehensive evaluation of the environmental sustainability of the new procedure. The energy economy factor (ϵ) was calculated

as:

$$\epsilon = \frac{Y_1 \cdot Y_2}{T_1 \cdot t_1 + T_2 \cdot t_2}$$

where Y_1 is the percentage mass recovery of polymer after the pretreatment, equal to 0.98, Y_2 is the TPA yield of the hydrolysis reaction, T_1 and t_1 indicate the temperature (in Celsius degrees) and duration (in minutes) of the pretreatment, T_2 and t_2 those of the alkaline hydrolysis. The environmental factor ($E = \frac{\text{mass of } waste}{\text{mass of } product}$) was modified into:

$$E = \frac{x_1 \frac{m_{solvent1}}{m_{PET}} + x_2 \frac{m_{solvent2}}{m_{PET}} + \frac{m_{NaOH}}{m_{PET}}}{Y_1 \cdot Y_2 \cdot \frac{M_{TPA}}{M_{PET}}}$$

where x_1 and x_2 respectively represent the mass percentages of non-recyclable solvent in the pretreatment step (PC) and the depolymerization step (water), while $m_{solvent1}$ and $m_{solvent2}$ denote their corresponding masses. Note that $m_{solvent2}$ includes both water retained in the wet PET, as determined by TGA, and the water externally added for the hydrolysis reaction (3 mL per g of PET). M_{TPA} and M_{PET} are the molecular weights of TPA (166.1 g/mol) and the repeating unit of PET (192.2 g/mol), respectively. Following the approach adopted in most studies, x_1 and x_2 were set to 0.1, reflecting a typical solvent loss of 10% during industrial-scale distillation [6]. The environmental energy impact factor (ξ) results from the combination of the first two factors as:

$$\xi = \frac{E}{\epsilon}$$

The best processes would tend to present low values of E, high values of ϵ, and then low values of ξ.

S2.3 Techno-economic analysis (TEA)

A techno-economic analysis (TEA) was carried out based on typical literature procedure [7, 8]. The operating cost was evaluated as the sum of different contributions:

$$\text{Operating cost (US\$/h)} = \text{C}_{\text{raw materials}} + \text{C}_{\text{utilities}} + \text{C}_{\text{labor}} + \text{C}_{\text{disposal}}$$

The specific operating cost was calculated as:

$$\text{Specific operating cost (US\$/t PET)} = \frac{\text{Operating cost (US\$/h)}}{\text{PET inlet (t/h PET)}}$$

where PET inlet is considered equal to 1 t h^{-1} PET.
To perform the analysis, a block diagram (Figure S2) was developed based on the experimental laboratory process. The process starts from PET waste, which is ground and dried to obtain clean flakes suitable for dissolution. In parallel, PC is heated to be used as the dissolution medium. In the dissolution unit, dried PET flakes are solubilized in hot PC to form a homogeneous PET-PC solution. This solution undergoes temperature-induced phase separation (TIPS) by quenching with water. The resulting suspension is processed through a liquid–solid (L–S) separation and washing step, yielding a PET-rich solid fraction (wet PET) which is recovered, and a solvent–water phase which is sent to distillation. Distillation allows recovery and recycle of PC and water, with a

small purge to prevent impurity buildup. The wet PET fraction is treated with aqueous NaOH in the alkaline hydrolysis step, where PET is converted to Na_2TPA and EG. The resulting raw liquid solution passes through a purification stage to remove impurities, affording a clean liquid solution. In the subsequent acidification and L–S separation, the purified solution is treated with H_2SO_4 to protonate Na_2TPA, precipitating TPA as a crystalline solid, the primary product. The liquid effluent from this step, containing H_2SO_4, Na_2SO_4, EG, and water, is further processed by separation operations. Solid Na_2SO_4 is isolated as a byproduct, while the remaining water–EG stream is directed to a final distillation, where EG is recovered as a secondary product and water is recycled to earlier process stages. Overall, the flowsheet enables complete conversion of PET waste into its molecular precursors, TPA and EG, with additional recovery of Na_2SO_4 as a byproduct. The recycled water stream from EG separation is fully valorized within the process. Specifically, it serves for preparing the NaOH solution used in alkaline hydrolysis, for purification of the hydrolyzed solution, and for the final washing step to remove residual glycol. This complete internal reuse of water enables zero-liquid-discharge operation, ensuring that all process streams are either recovered or recycled in line with circular-economy and sustainability principles.

For the techno-economic evaluation, the costs of raw materials (make-up water, make-up PC, H_2SO_4, NaOH), high-pressure steam (HPS) used for heating and distillation duties, and labor for plant operation were considered (Table S2).

Material balance results are expressed in $\mathrm{t\,h^{-1}}$ for each stream. From 1.00 $\mathrm{t\,h^{-1}}$ of PET, 0.81 $\mathrm{t\,h^{-1}}$ of TPA is produced (Figure S3).

The operating cost analysis highlights the main cost contributors within the system. The PC make-up, NaOH, and H_2SO_4, together with labor, represent the major cost items, while water make-up, water treatment, and high-pressure steam (HPS) have a smaller impact (Figure S4). The total operating cost amounts to US\$612 $\mathrm{h^{-1}}$.

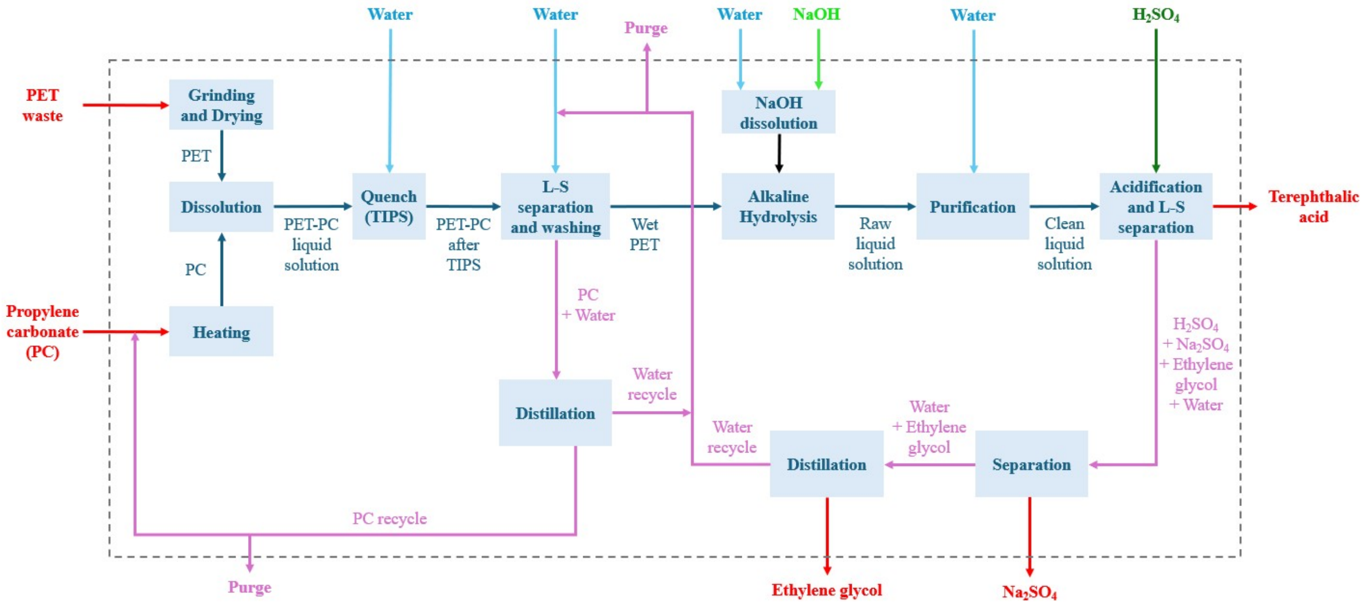

Figure S2: Block diagram of the techno-economic analysis based on the experimental laboratory process.

Table S2: Prices of raw materials, disposal, utilities, and labor used in the techno-economic analysis.

	Parameter	Price	Units	Reference
**Raw materials**	Water make-up	3	US$/t	[8]
	PC make-up	910	US$/t	[9]
	H_2SO_4	125	US$/t	[10]
	NaOH	300	US$/t	[11]
**Disposal**	Water treatment (WT)	2	US$/t	[12]
**Utilities**	High-pressure steam (HPS)	5.66	US$/GJ	[13]
**Labor**	Average salary	57	kUS$/year	[7]

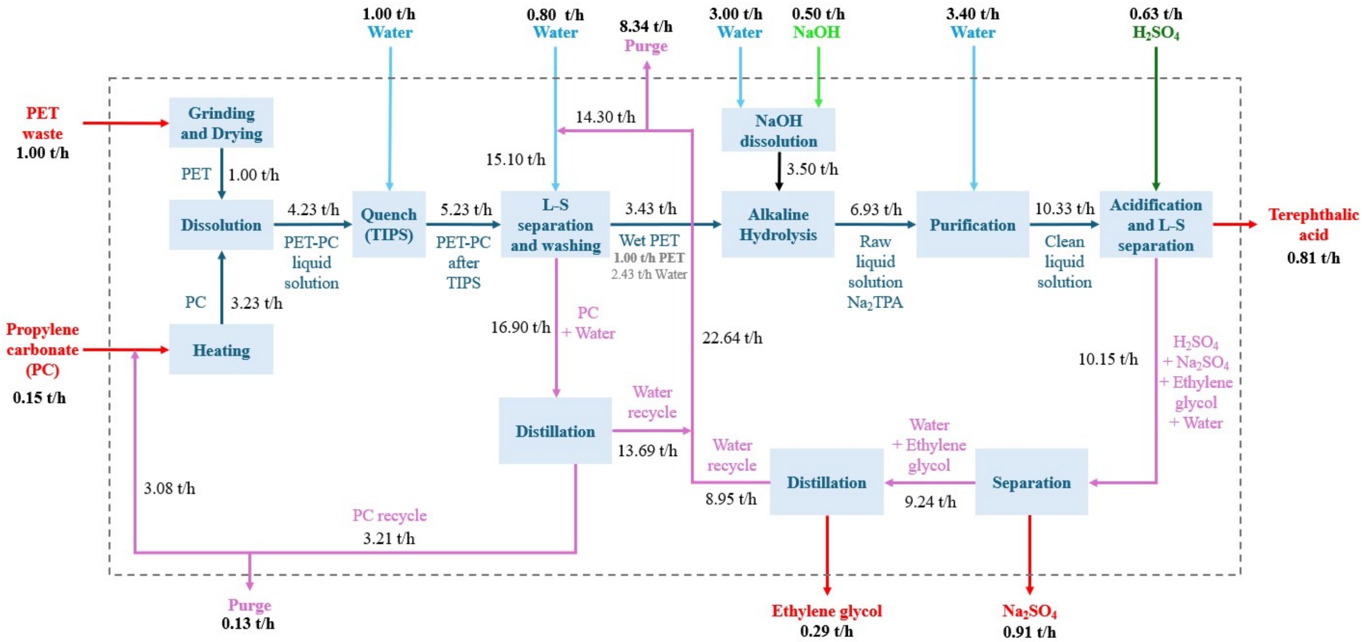

Figure S3: Block diagram and mass balance results from process simulation.

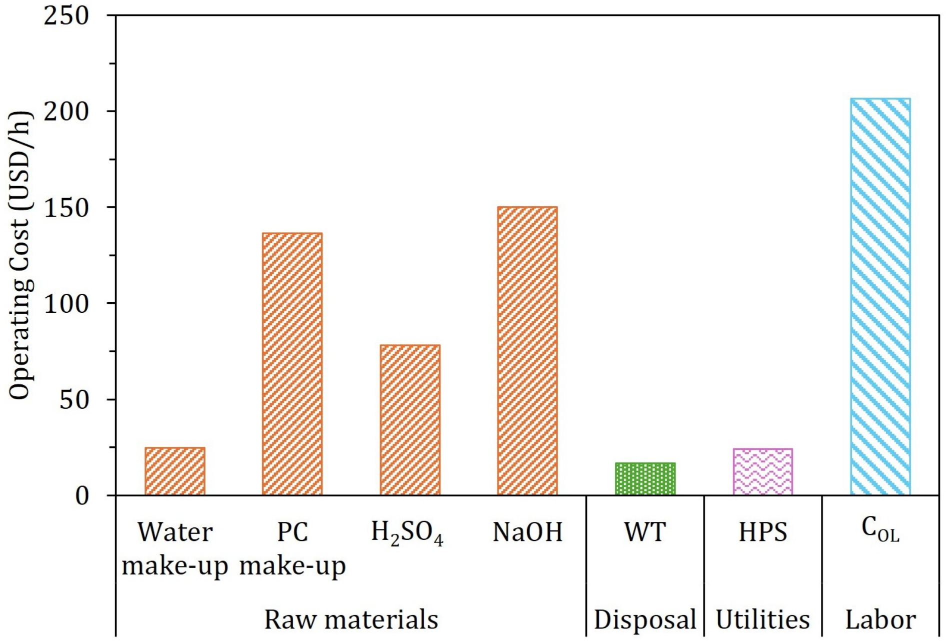

Figure S4: Operating cost (US\$ h^{-1}) of each process parameter in the techno-economic analysis.

3 Supplementary Tables

Table S3: Sustainability scores for solvents used in alkaline hydrolysis methods. Data for the categories of health (H), safety (S), environment (E), and waste disposal (W) are taken from the GSK solvent sustainability guide [14]. The final composite score, $G = \sqrt[4]{H \times S \times E \times W}$, reflects the overall greenness of the solvent, according to the following numerical scale [15]:
highly sustainable solvent ($G \geq 7$); solvent with a limited number of sustainability issues ($G = 5 - 6$); solvent that should be avoided ($G \leq 4$).

**Solvent**	**Health (H)**	**Safety (S)**	**Environment (E)**	**Waste Disposal (W)**	**Composite Score (G)**
Propylene carbonate	10	10	10	5.9	8.8
Ethylene carbonate	8.4	9.4	10	5.2	8.0
γ-valerolactone	5.3	9.5	7.7	8.4	7.6
Methanol	4.9	7.1	8.4	4.0	5.8
Ethanol	8.9	7.7	6.7	4.2	6.6
Ethylene glycol	8.4	10	8.9	5.6	8.1
Dichloromethane	5.3	6.3	6.9	3.0	5.1

Table S4: Temperature of degradation onset ($T_{5\%}$), temperature of maximum degradation rate (T_{max}), and mass fraction of residue at 600 °C for virgin PET and dried PET, obtained from TGA.

**Sample**	**$T_{5\%}$ (°C)**	**T_{max} (°C)**	**Residue at 600 °C (%)**
Virgin	404	433	9.6
Dried	406	434	11.9

Table S5: GPC parameters for virgin PET and dried PET.

**Sample**	**M_p[1] (Da)**	**M_n[b] (Da)**	**M_w[c] (Da)**	**M_z[d] (Da)**	**M_v[e] (Da)**	**PDI[f]**
Virgin	61600	28300	91000	233500	208900	3.2
Dried	50400	23500	66000	145800	132300	2.8

Table S6: WAXD analysis of virgin and dried PET. For each sample, the table reports the relative position and area of the fitted peaks, as well as the overall crystallinity and average lamellar thickness along the 001 chain direction (D_{001}).

Sample	Peak position (°)	Peak area	Cristallinity	D_{001} (nm)
Virgin	16.45	80694.8	42%	3.5
	17.68	92173.3		
	21.67	17127.2		
	22.97	147301.8		
	26.08	310050.1		
	28.02	29403.3		
	32.74	23096.4		
	21.81 (amorphous)	970580.6		
Dried	16.23	162986.9	46%	2.5
	17.58	165987		
	21.16	31749.6		
	22.61	238868.3		
	24.65	114467.5		
	26.10	396708		
	27.90	68139.1		
	32.41	48824.1		
	21.29 (amorphous)	1425751		

Table S7: DSC results for virgin and dried PET: first melting endotherm peak temperature (T_{m1}); second melting endotherm peak temperature (T_{m2}); total melting enthalpy (ΔH_m); peak temperature of crystallization from the melt (T_c); enthalpy of crystallization from the melt (ΔH_c); peak temperature of cold crystallization (T_{cc}); enthalpy of cold crystallization (ΔH_{cc}).

Sample	1st heating			Cooling		2nd heating			
	T_{m1} (°C)	T_{m2} (°C)	ΔH_m (J/g)	T_c (°C)	ΔH_c (J/g)	T_{cc} (°C)	ΔH_{cc} (J/g)	T_m (°C)	ΔH_m (J/g)
Virgin	237.7	247.7	-54.7	159.3	14.6	161.4	11.6	245.9	-28.6
Dried	241.5	247.6	-53.1	179.5	36.5	–	–	247.8	-36.5

Table S8: Experiments of alkaline hydrolysis carried out on virgin, wet, and dried PET. The reaction conditions are: 5 g PET, 2.5 g NaOH, 15 mL H_2O. All experiments are performed in triplicate, and the results are reported as mean value ±SD.

Exp #	Sample	T (°C)	time (min)	PET conversion (%)	TPA yield (%)
1	Virgin PET	90	10	9±2	9±2
2	Wet PET	90	10	99.2±0.5	96.1±0.2
3	Dried PET	90	10	33±4	24±4
4	Virgin PET	90	960	97.6±1.3	96±2
5	Dried PET	90	180	98.5±0.9	95.7±1.4
6	Wet PET (EC)[1]	90	10	98.3±0.7	95±2

[1] Sample prepared using ethylene carbonate (EC) as disssolution solvent in the pre-treatment.

Table S9: Experiments of alkaline hydrolysis carried out on wet PET at various temperatures. The reaction conditions are: 1 g PET, 0.5 g NaOH, 3 mL H_2O. All experiments are performed in triplicate, and the results are reported as mean value ±SD.

Exp #	Sample	T (°C)	time (min)	PET conversion (%)	TPA yield (%)
7	Wet PET	25	5	46±3	39±3
8	Wet PET	25	15	70±2	60±3
9	Wet PET	25	30	85±2	73±3
10	Wet PET	25	60	95±2	96±2
11	Wet PET	25	120	98.1±0.8	94±3
12	Wet PET	40	2.5	40±2	29±2
13	Wet PET	40	5	64.0±1.0	54.2±1.3
14	Wet PET	40	15	87±2	79.4±1.1
15	Wet PET	40	30	96.0±0.9	93.7±0.4
16	Wet PET	50	2.5	62±2	57±2
17	Wet PET	50	5	77.4±1.2	66±2
18	Wet PET	50	10	90.3±1.4	82.2±1.1
19	Wet PET	50	15	95±2	91±2
20	Wet PET	60	1	54±4	48±4
21	Wet PET	60	2.5	73±3	66±3
22	Wet PET	60	5	85±2	78±2
23	Wet PET	60	10	94.2±1.3	88±2
24	Wet PET	70	5	93±2	86±2
25	Wet PET	80	5	98±2	93.9±1.3
26	Wet PET	90	1	83±2	72±3
27	Wet PET	90	2.5	93±2	83±3
28	Wet PET	90	5	99.1±0.5	95.0±1.1
29	Wet PET	90	10	99.1±0.5	96.2±1.1
30	Wet PET	90	15	98.7±0.3	96.4±0.9
31	Wet PET (1 g NaOH)[1]	25	15	95±2	91±2

[1] Depolymerization performed using an equal amount of PET and NaOH (1 g).

Table S10: Alkaline hydrolysis of pre and post-consumer PET products. The reaction conditions are: 5 g PET, 2.5 g NaOH, 15 mL H_2O, 10 min at 90 °C. All samples are pre-treated using PC as dissolution solvent. All experiments are performed in triplicate, and the results are reported as mean value ±SD. The values in parenthesis refer to the materials without pre-treatment.

Alkaline hydrolysis of pre and post-consumer products					
**Sample**	**Photo**	**Recovered TPA**	**Dissolution time in PC (min)**	**PET Conversion (%)**	**TPA Yield (%)**
PET pellets (Poliplast srl)	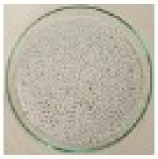	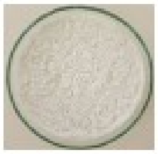	25	99.2±0.5 (0)	96±2 (0)
Coca-cola Bottle (100% rPET)	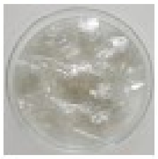	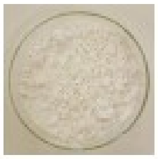	10	97.2±1.2 (1.2±0.4)	94.4±1.4 (0)
Blue Bottle	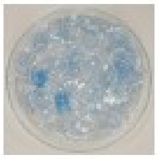	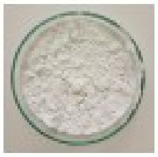	10	99.2±0.5 (3.1±1.3)	94±2 (0)
Green Bottle	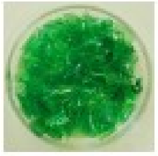	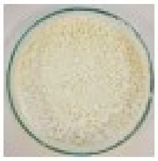	10	98.2±1.1 (1.2±0.6)	95±2 (0)
White Textile (online purchased)	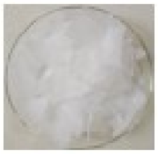	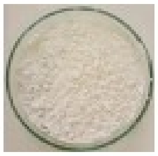	10	96.7±1.4 (7±2)	91±2 (4±2)
Green Textile (medical gown)	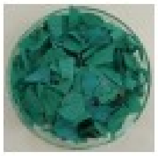	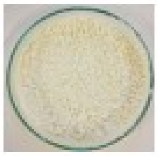	10	90±2 (9±3)	85±2 (0)
Shredded Waste (recycling plant)	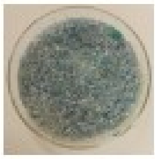	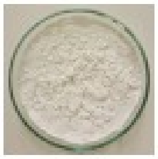	25	98.3±1.1 (0)	95.2±1.1 (0)

Table S11: Alkaline hydrolysis of mixed waste. The reaction conditions are: 5 g PET, 2.5 g NaOH, 15 mL H_2O, 10 min at 90 °C. All samples are pre-treated using PC as dissolution solvent. All experiments are performed in triplicate, and the results are reported as mean value ±SD. The values in parenthesis refer to the materials without pre-treatment.

Alkaline hydrolysis of mixed post-consumer products					
**Sample**	**Photo**	**Recovered TPA**	**Dissolution time in PC (min)**	**PET Conversion (%)**	**TPA Yield (%)**
Blue bottle + PE cup	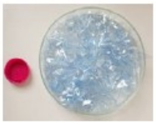	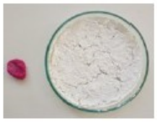	10	99.2±0.6 (2.0±1.1)	96±2 (0)
Transparent box + PP cup	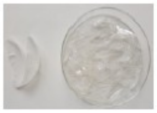	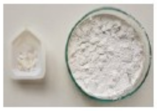	10	97.2±1.2 (0)	90.1±1.4 (0)

Process	Reaction	Main Products	Operating Conditions	Industrial examples	Advantages	Main limitations	Estimated Energy Consumption (MJ/kg PET)	Energy Cost (US$/t PET)
Glycolysis [6, 16, 17, 18]	Transesterification of PET with EG	BHET + oligomers	180–240 °C<br>1–3 bar	Eastman<br>Loop<br>Ioniqa	Established technique<br>High BHET purity	Requires product purification	8–12	26
Methanolysis [6, 19, 20]	Methanol cleavage → DMT + EG	DMT + EG	200–280 °C<br>20–40 bar	Eastman<br>Teijin	High DMT purity<br>Good integration with traditional processes	High P and T<br>More energy-intensive	15–20	61
Alkaline / neutral hydrolysis [6, 20, 21]	Cleavage with H_2O or NaOH → TPA + EG	TPA + EG	200–250 °C<br>10–20 bar	GR3N (microwave)<br>Sabic	Very pure TPA<br>No organic solvents	Need for neutralization<br>Wastewater	10–15	44
Hydrolysis with microwave [19, 21, 22]	Microwave-assisted alkaline hydrolysis	TPA + EG	180–200 °C<br>1–5 bar	GR3N – DEMETO	Fast reactions<br>Mild conditions<br>Low environmental impact	Industrial expanding technology	5–8	55
Enzymatic depolymerization [19, 22, 23]	Enzyme cleavage (PETase, MHETase)	TPA + EG	50–70 °C<br>pH 7–8	Carbios<br>Protein Evolution	Mild conditions<br>High selectivity	Enzyme cost and stability<br>Need for amorphization ’	2–6	18
Aminolysis / Ammonolysis [18, 24, 25]	Reaction with NH_3 or amines	Terephthalamides + EG	150–220 °C	BASF (R&D)<br>IIT	Upcycling to valuable products	Does not regenerate virgin PET	10–13	—
Integrated / hybrid processes [6, 20, 21, 22]	Combined (e.g., microwave + hydrolysis catalysis + purification)	TPA + EG/BHET	Variables	Loop<br>Eastman<br>GR3N	High efficiency<br>High purity	Greater plant complexity	6–10	—
This work *(alkaline hydrolysis)*	Cleavage with NaOH → TPA + EG	TPA + EG	25–90 °C<br>1 bar	—	Ultrafast reaction<br>Low energy demand<br>Simple setup	To be validated at scale	3.22	36.18

Table S12: Comparison of PET chemical recycling processes: main features, operating conditions, and estimated energy demand.

4 Supplementary Figures

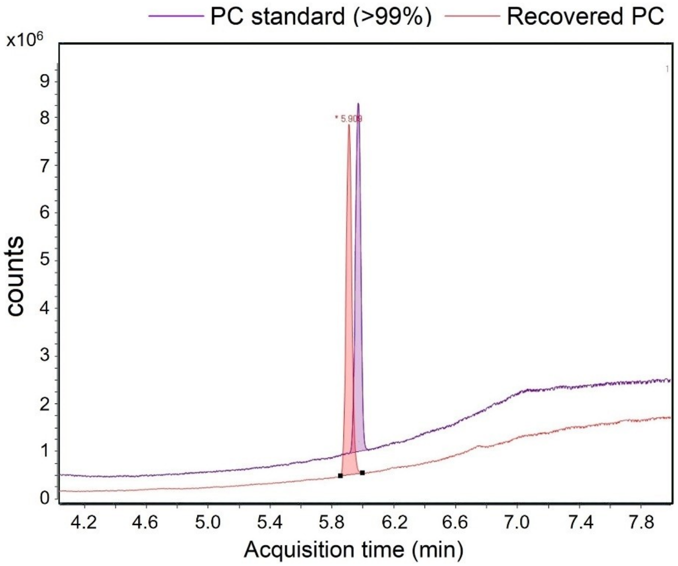

Figure S5: GC-MS chromatogram of a high-purity PC standard and of PC isolated after PET pre-treatment, both diluted at 1000 ppm in $CHCl_3$.

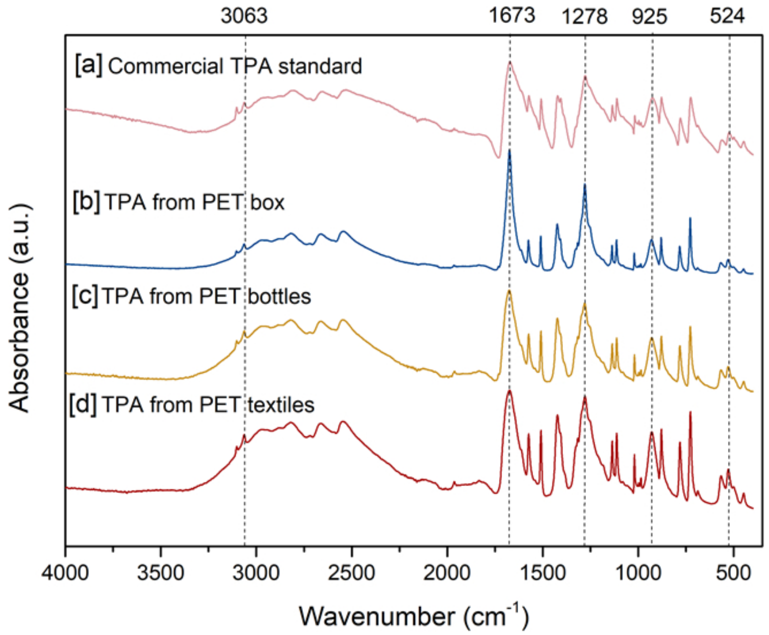

Figure S6: ATR-FTIR spectra of TPA recovered from different sources of post-consumer PET waste.

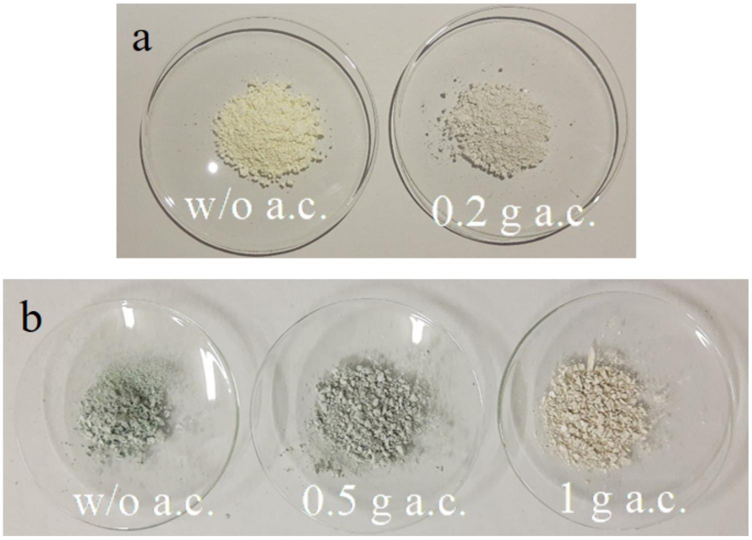

Figure S7: Decolorization of TPA obtained from (a) green bottle and (b) green textiles using different amounts of activated carbon (a.c.). The reaction conditions are: 1 g PET, 0.5 g NaOH, 3 mL H_2O, 10 min at 90 °C. At the end of the reaction, a.c. is added to the mixture along with 20 mL of water, followed by continuous stirring for an additional 20 min at 90 °C.

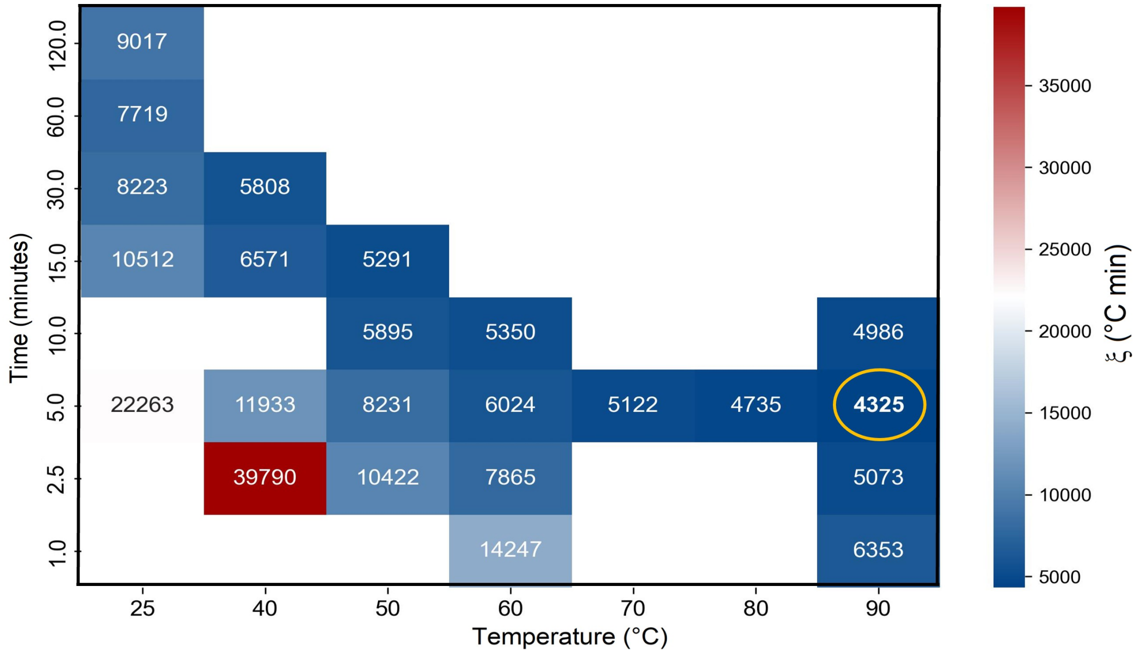

Figure S8: Heatmap of the environmental energy impact factor, ξ, calculated for the alkaline hydrolysis of wet PET at different reaction times and temperatures. The reaction conditions are: 1 g PET, 0.5 g NaOH, 3 mL H_2O.

References

[1] C. A. Massa, S. Capaccioli, A. Manariti, M. Bertoldo, "Study of the Cold Crystallization of Poly(ethylene terephthalate) at the Air Interface by ATR Spectroscopy" *Eur. Polym. J.* **2014**, *60*, 286.

[2] Z.-G. Wang, B.S. Hsiao, B.X. Fu, L. Liu, F. Yeh, B.B. Sauer, H. Chang, J. M. Schultz, "Correct Determination of Crystal Lamellar Thickness in Semicrystalline Poly(ethylene terephthalate) by Small-Angle X-Ray Scattering" *Polymer* **2000**, *41*, 1791.

[3] A. M. Haregewoin *et al.*, "A combined experimental and theoretical study of surface film formation: Effect of oxygen on the reduction mechanism of propylene carbonate" *Journal of Power Sources* **244**, *318* (2013).

[4] B. Hanschmann, "Precipitation of Polypropylene and Polyethylene Terephthalate Powders Using Green Solvents via Temperature and Antisolvent-Induced Phase Separation" *Advances in Polymer Technology* **2023**, *1* (2023).

[5] M. Murase, D. Nakamura, "Hansen Solubility Parameters for Directly Dealing with Surface and Interfacial Phenomena" *Langmuir* **39**, *10475* (2023).

[6] E. Barnard, J. J. Rubio Arias, W. Thielemans, "Chemolytic Depolymerisation of PET: a Review" *Green Chem.* **2021**, *23*, 3765.

[7] "Ludwig's Applied Process Design for Chemical and Petrochemical Plants" **2007**, edited by A. Kayode Coker, *Chapter 6–Mechanical Separations 1*, 371.

[8] R. Turton, R. C. Bailie, W. B. Whiting, "Analysis, synthesis, and Design of Chemical Processes" **2008**, Prentice Hall, Upper Saddle River.

[9] IMARC Group, "Propylene Carbonate Pricing Report 2024: Price Trend, Chart, Market Analysis, News, Demand, Historical and Forecast Data" *Report ID: SR112025A22397*, **2024**, https://www.imarcgroup.com/propylene-carbonate-pricing-report (accessed October 2, 2025).

[10] IMARC Group, "Sulfuric Acid Prices, Trend, Chart, Demand, Market Analysis, News, Historical and Forecast Data Report 2025 Edition" *Report ID: SR112025A23214*, **2025**, https://www.imarcgroup.com/sulfuric-acid-pricing-report (accessed October 2, 2025).

[11] BusinessAnalytiq, "Sodium hydroxide price index, Prices Indexes" **2025**, https://businessanalytiq.com/procurementanalytics/index/sodium-hydroxide-price-index/ (accessed October 2, 2025).

[12] Y. Lyu, H. Ye, Z. Zhao, J. Tian, L. Chen, "Exploring the cost of wastewater treatment in a chemical industrial Park: Model development and application" *Resources, Conservation & Recycling* **2020**, *155*, 104663.

[13] M. Salimi, M. Amidpour, M.A. Moradi, M. Hajivand, E. Siahkamari, M. Shams, "Technical-Economic Analysis of Energy Efficiency Solutions for the Industrial Steam System of a Natural Gas Processing Plant" *Sustainability* **2023**, *15*, 14995.

[14] C. M. Alder *et al.*, "Updating and Further Expanding GSK's Solvent Sustainability Guide" *Green Chem.* **2016**, *18*, 3879.

[15] C. Larsen, P. Lundberg, S. Tang, J.Ràfols-Ribé, A. Sandström, E. Mattias Lindh, J. Wang, L. Edman, "A Tool for Identifying Green Solvents for Printed Electronics" *Nat. Commun.* **2021**, *12*, 4510.

[16] M. Gabrič, Ž. Lavrič, M. Schwiderski, L. Marc, E. Temmel, M. Grilc, B. Likozar, "Polyethylene terephthalate glycolysis: kinetic modeling and validation" *Polymers* **2025**, *17*, 2246.

[17] A. Enache, I. Grecu, P. Samoilă, "Polyethylene terephthalate (PET) recycled by catalytic glycolysis: a bridge toward circular economy principles" *Materials* **2024**, *17*, 2991.

[18] Z. Jia, L. Gao, L. Qin, J. Yin, "Chemical recycling of PET to value-added products" *RSC Sustainability* **2023**, *1*, 2135.

[19] Guo, Z., Wu, J., Wang, J., "Chemical degradation and recycling of polyethylene terephthalate (PET): a review" *RSC Sustainability* **2025**, *3*, 2111.

[20] K. Ragaert, L. Delva, K. Van Geem, "Mechanical and chemical recycling of solid plastic waste" *Waste Management* **2017**, *69*, 24.

[21] J. Volmajer Valh, D. Stopar, I. Selaya Berodia, L. Fras Zemljič, "Economical Chemical Recycling of Complex PET Waste in the Form of Active Packaging Material" *Polymers* **2022**, *14*, 3244.

[22] D. Peti, J. Dobránsky, P. Michalík, "Recent Advances in Polymer Recycling: A Review of Chemical and Biological Processes for Sustainable Solutions" *Polymers* **2025**, *17*, 603.

[23] L. Pastor, K. Schell, S. Göbbels, F. Contreras, M. Bienstein, G.Jäger, U. Schwaneberg, L. Reisky, "Identification of urethanases for biocatalytic recycling of toluene diisocyanate- and methylene diphenyl diisocyanate-based polyurethanes" *ChemSusChem* **2025**, *18*, e202500662.

[24] R. Kuang, Q. Duan, X. Kuang, "Conversion of waste pet into triazines-incorporated polyols and flame-retardant polyurethane foams" *Physica Scripta* **2023**, *98*, 105905.

[25] J. Payne, G. Kociok-Köhn, E. A. C. Emanuelsson, M. D. Jones, "Zn(II)- and Mg(II)-complexes of a tridentate {ONN} ligand: application to poly(lactic acid) production and chemical upcycling of polyesters" *Macromolecules* **2021**, *54*, 8453.
